# The effect of the *Rht1* haplotype on Fusarium head blight resistance in relation to type and level of background resistance and in combination with *Fhb1* and *Qfhs.ifa-5A*

**DOI:** 10.1007/s00122-022-04088-x

**Published:** 2022-04-09

**Authors:** Maria Buerstmayr, Hermann Buerstmayr

**Affiliations:** Department of Agrobiotechnology, Institute of Biotechnology in Plant Production, University of Natural Resources and Life Sciences Vienna, Konrad Lorenz Str. 20, 3430 Tulln, Austria

## Abstract

**Key message:**

The effect of the *Rht1*-genes on FHB resistance depends on anther extrusion and level of background resistance. *Qfhs.ifa-5A* increases resistance and anther extrusion as efficiently as semi-dwarfing alleles decrease it.

**Abstract:**

The semi-dwarfing reduced height alleles *Rht-D1b* and *Rht-B1b* have been deployed in modern wheat cultivars throughout the world, but they increase susceptibility to Fusarium head blight (FHB). Here, we investigated the impact of the *Rht1* genes on anther retention (AR) in relation to FHB resistance using four different sets of near-isogenic lines (NILs) with contrasting levels and types of background FHB resistance. NILs were evaluated for FHB severity, plant height and AR in three greenhouse and three field trials using artificial spray inoculation. *Rht-B1b* and *Rht-D1b* alleles increased AR and FHB susceptibility in all genetic backgrounds. The magnitude of the effects differed between NIL groups. Increased FHB susceptibility largely followed increased AR. Differences in FHB susceptibility between tall and dwarf haplotypes were largest in the NIL group with the highest changes in AR. In the most resistant NIL group, dwarfed lines had only slightly higher AR than tall lines and maintained good resistance, while both tall and dwarf lines had high levels of retained anthers in the most susceptible NIL group. We further investigated the effect of the major Fusarium resistance QTL *Fhb1* and *Qfhs.ifa-5A* in combination with the *Rht1* genes. *Qfhs.ifa-5A* enhanced anther extrusion in tall as well as semi-dwarf haplotypes, whereas *Fhb1* did not affect AR. *Qfhs.ifa-5A* supported FHB resistance more efficiently than *Fhb1* in lines that were more responsive to AR, while both *Fhb1* and *Qfhs.ifa-5A* were equally efficient in NILs that had high background resistance and low response to AR.

**Supplementary Information:**

The online version contains supplementary material available at 10.1007/s00122-022-04088-x.

## Introduction

Fusarium head blight (FHB), primarily caused by members of the *Fusarium graminearum* species complex, is a highly destructive disease that affects wheat and small grains worldwide (Parry et al. 1995). Prolonged warm and humid conditions around flowering can cause severe FHB outbreaks that can drastically reduce grain yield and quality (McMullen et al. 2012). FHB is furthermore of great concern for food and feed safety as mycotoxins produced by *Fusarium* pathogens accumulate in infected grains and pose a health risk to humans and animals (Pestka 2010).

FHB resistance is a highly complex quantitative trait controlled by a multitude of genetic and non-genetic factors. Pheno-morphological traits, particularly plant height, spike architecture and flowering related traits, modify disease response (Buerstmayr et al. 2020; Zhu et al. 1999). Many studies have observed higher susceptibility to fungal entry (type 1 resistance) and reduced overall ‘field resistance’ of short-straw cultivars compared to tall ones when tested in the field. Of the QTL mapping studies that reported on both plant height and FHB resistance, 40% of the plant height QTL overlapped with QTL for type 1 resistance or overall ‘field resistance’ (Buerstmayr et al. 2020). The observed association between plant height and FHB resistance may depend on several factors that affect the likelihood of infection and influence the microclimate at spike height. The pathogen overwinters saprophytically on plant debris that constitutes the primary inoculum for upcoming crops. FHB infection starts inside the florets with the plants being most sensitive around flowering (Parry et al. 1995). Spores enter the spikelets during anthesis when florets open or via degenerated anthers that are partially trapped between the glumes (Pugh 1933). Shorter plants are thus at greater risk for getting infected by rain-splash-dispersed spores (Jenkinson and Parry 1994). Heads on taller plants dry quickly, as they are exposed to wind and sun. In contrast, shorter plants are more affected by soil humidity and dew, where the dense canopy structure with the flag leaves close to the heads may reduce air circulation, thereby increasing relative humidity and temperature around spikes that favor infection and disease development (Jones et al. 2018).

The significance of anthers and their effect on FHB resistance has long been known (Pugh 1933) and has recently regained attention. Fully extruded anthers (Steiner et al. 2019) and complete cleistogamous flowering (Kubo et al. 2010) are associated with FHB resistance, while susceptibility increases with the proportion of retained anthers or anthers partially trapped between palea and lemma (Buerstmayr and Buerstmayr 2015; Kubo et al. 2013). Among the mapping studies that collected data on anther retention (AR) or anther extrusion (AE), plant height and FHB resistance, 60% of the QTL for AR/AE coincided with FHB resistance QTL, whereas overlap of QTL for plant height and AR/AE were rare, indicating that AR/AE and plant height are largely independently genetically controlled (Buerstmayr et al. 2020).

The implementation of the reduced height (*Rht*) alleles in wheat and rice breeding since the 1960s was key elements of the ‘Green Revolution’ (Borlaug 1968). Plants with the semi-dwarfing *Rht1* alleles are shorter, have an improved harvest index combined with higher grain yield and better resistance to lodging. This facilitated an intensification of farming systems and led to a substantial increase in grain yield worldwide (Hedden 2003). To date in many wheat growing regions, modern wheat cultivars and breeding lines frequently possess one of the homeolog mutant semi-dwarfing alleles *Rht-B1b* or *Rht-D1b* located on the short arm of chromosomes 4B and 4D, respectively (Evans 1998; Würschum et al. 2015). The *Rht1* genes encode DELLA proteins, which belong to a plant-specific family of transcriptional regulators and are involved in the signal transduction pathway of the phytohormone gibberellin (GA) with the wild-type alleles being sensitive to GA (Achard et al. 2006). GA mediates the degradation of the growth repressing DELLA proteins allowing the plants to grow tall (Peng et al. 1997). The mutant *Rht-B1b* and *Rht-D1b* alleles have similar nucleotide substitutions in the N-terminal DELLA domain that result in premature stop codons. Due to translational reinitiation in the main open reading frame N-terminally truncated DELLA proteins are produced, which are resistant to GA-mediated degradation. This causes a lower reduction of the growth repressing DELLA proteins and plants remain short (Peng et al. 1999; Van De Velde et al. 2021). The *Rht1* genes influence a multitude of agronomically relevant traits that contribute to yield and grain quality, for instance coleoptile size and seed emergence, grain number per spikelet, seed size, protein content and grain yield (Acuña et al. 2001; Flintham et al. 1997). The semi-dwarfing alleles have a negative effect on anther extrusion (Boeven et al. 2016; Buerstmayr and Buerstmayr 2016; Lu et al. 2013; Muqaddasi et al. 2017). The combination of reduced height and high number of retained anthers particularly negatively impacts FHB resistance. This may explain the observed high effect of the *Rht1* loci on type 1 and overall field resistance in almost all mapping populations that segregated for the *Rht1* alleles (Buerstmayr et al. 2020; Xu et al. 2020; Zhu et al. 2021).

The major FHB resistance QTL *Fhb1* and *Qfhs.ifa-5A* are the most extensively studied and best-validated FHB resistance QTL, and they have been successfully implemented in resistance breeding programs (Buerstmayr et al. 2020; McMullen et al. 2012; Steiner et al. 2017). *Qfhs.ifa-5A* confers resistance to fungal entry (type 1 resistance) (Buerstmayr et al. 2003) and increases anther extrusion (Steiner et al. 2019), while *Fhb1* acts against fungal spreading within the spike (type 2 resistance) (Waldron et al. 1999).

In our work, we specifically investigated the effect of the *Rht1* genes on AR and on FHB resistance in *i)* four genetic backgrounds. These genetic backgrounds differed in types and levels of FHB resistance and *ii)* we investigated the effect of the major FHB resistance QTL *Fhb1* and *Qfhs.ifa-5A* in combination with the *Rht1* genes. For this, we generated near-isogenic lines (NILs) of spring wheat lines ‘CM-82036’ (superior type 1 and type 2 resistance, carrier of *Fhb1* and *Qfhs.ifa-5A*), ‘Remus-NIL1’ (moderate type 1 and moderate type 2 resistance, carrier of *Fhb1* and *Qfhs.ifa-5A*), ‘FRxRE’, a double haploid line selected from a Frontana-by-Remus cross (high type 1 resistance), and ‘Michael’ (susceptible) that were homozygous for either one of the two semi-dwarfing alleles (*Rht-D1b* or *Rht-B1b*) or the wild-type alleles *Rht-B1a/Rht-D1a.* We furthermore developed and analyzed CM-82036 and Remus NILs with contrasting allele status at *Rht-B1*, *Rht-D1*, *Fhb1,* and *Qfhs.ifa-5A.*

## Material and Methods

### Development of NIL groups

#### Development of *Rht1* NIL groups Remus-NIL1 (RE-NIL1), CM-82036 (CM), Frontana-by-Remus (FRxRE) and Michael (MI)

*Rht-D1b* (donor cultivar Monsun) or *Rht-B1b* (donor cultivar Bobwhite) were each five times back-crossed to spring wheat recipient lines ‘Remus-NIL1’, ‘CM-82036’, ‘FRxRE’ or ‘Michael’ leading to an expected recovery of ~ 99% for the recipient genome. All four recipient lines possess the tall wild-type alleles (*Rht-B1a*/*Rht-D1a*) of the *Rht1* genes but differ in their resistance to FHB. ‘CM-82036’ originates from the cross Sumai 3/Thornbird-S. CM-82036 has outstanding type 1 and type 2 resistance and contains the FHB resistance QTL *Fhb1* and *Qfhs.ifa-5A* (Buerstmayr et al. 2002; 2003). Remus-NIL1 is near isogenic to Remus and possesses moderate type 1 and type 2 resistance. It contains the resistance alleles of *Fhb1* and *Qfhs.ifa‐5A* embedded in the susceptible background of the cultivar ‘Remus’ (Schweiger et al. 2013). Line FRxRE was selected from a doubled haploid population developed from a cross of Frontana and Remus (Steiner et al. 2004). FRxRE shows good type 1 resistance but lacks type 2 resistance. Michael is a high-yielding commercial spring wheat released by Saatzucht Bauer GmbH & Co KG, Germany. Michael is highly susceptible to FHB.

*Rht-B1* and *Rht-D1* markers as described by Ellis et al. (2002) or allele-specific KASP assays retrieved from the Cerealsdb database (www.cerealsdb.uk.net/cerealgenomics/CerealsDB/Excel/MAS_data_May_2013.xls) were used to ensure the transmission of the *Rht-D1b* and *Rht-B1b* allele through the five backcrossing cycles. Per backcross stream two heterozygous BC_5_F_1_ plants were selfed and plants homozygous for the dwarf or tall alleles at the *Rht1* genes were selected from BC_5_F_2_ plants. The thus-developed plant material comprises four *Rht1*-NIL groups defined by their recurrent parent (abbreviations for NIL groups: RE-NIL1, CM, FRxRE, MI), each containing five, two and two NILs of the *Rht1*-haplotypes *B1aD1a, B1bD1a*, and *B1aD1b,* respectively (Table 1).

**Table 1:** Number of NILs per *Rht1* haplotype of NIL groups Remus-NIL1 (RE-NIL1), CM-82036 (CM), Frontana by Remus derived DH line (FRxRE) and Michael (MI)

*Rht1* haplotype	RE-NIL1^a^	CM^a^	FRxRE	MI
*Rht-B1aD1a*^b^	5	5	5	5
*Rht-B1bD1a*^c^	2	2	2	2
*Rht-B1aD1b*^d^	2	2	2	2

^a^Carrier of the FHB resistance QTL *Fhb1* and *Qfhs.ifa-5A*

^b^Includes recurrent parent and tall NILs of crosses with Bobwhite and Monsun

^c^*Rht-B1b* donor is ‘Bobwhite’

^d^*Rht-D1b* donor is ‘Monsun’

#### Development of RE and CM QTL-by-Rht1 NILs

Each semi-dwarfing *Rht1*-NIL of group RE-NIL1 and CM were crossed with Remus or CM-NIL51, respectively. Remus and CM-NIL51 (near isogenic to CM) have the tall *Rht-B1a/Rht-D1a* haplotype and the susceptible alleles at *Fhb1* and *Qfhs.ifa-5A* (Schweiger et al. 2016). Per cross all possible QTL-by-Rht1 combinations were selected (Table 2) using allele-specific KASP assays for *Rht-B1, Rht-D1 and Fhb1* (Su et al. 2018) and SSR markers *gwm304* (Röder et al. 1998) and *wmc705* (Somers et al. 2004) that flank the *Qfhs.ifa-5A QTL* interval.

**Table 2:** Number of Remus (RE) and CM-82036 (CM) QTL-by-Rht1 NILs per FHB resistance QTL and *Rht1* haplotype combination

	FHB resistance QTL			
*Rht1* haplotype	*Fhb1* and *Qfhs.ifa-5A*	*Qfhs.ifa-5A*	*Fhb1*	No QTL
*Rht-B1aD1a*^a^	4	4	4	4
*Rht-B1bD1a*^b^	2	2	2	2
*Rht-B1aD1b*^c^	2	2	2	2

^a^Includes tall variants of crosses with Bobwhite and Monsun

^b^*Rht-B1b* donor is ‘Bobwhite’

^c^*Rht-D1b* donor is ‘Monsun’

### Fusarium inoculation and trait assessments

#### Green house experiments

Three greenhouse experiments were conducted with two replicates per genotype in January 2020 and in December 2020 and an un-replicated experiment in February 2021. Seeds of *Rht1*-NILs and recurrent parents were germinated in seedling starter trays on a mixture of recycled compost and sand and vernalized at 4 °C with a 12-h day/night light regime for 1 week. Twelve seedlings per NIL genotype were replanted into pots (21 cm diameter, 23 cm height) filled with 6.5 l of potting soil (75% heat-sterilized recycled compost, 23% peat, 2% silica sand) and transferred to the greenhouse.

In the greenhouse within each block, NILs for each NIL group and the respective recurrent parent were grouped together. Per NIL group semi-dwarfing and tall NILs were furthermore organized into subgroups whereby NILs within subgroups were fully randomized. This design allowed to adjust the distance between wheat heads and green house lamps during the inoculation and scoring period so that heads of dwarf and tall lines had the same distance of approximately 80 cm to the greenhouse lamps (Philips MASTER SON-T PIA Plus 400 W) and were therefore equally affected by radiant heat emitted by the lamps. Temperature in the greenhouse was set to 18/12 °C (day/night) from tillering to heading with 12–14 h of light. Shortly before anthesis, the greenhouse conditions were set at 22/18 °C (day/night) with a 16-h photoperiod at 15,000 lx, and settings were kept constant until the end of the experiments. *F. graminearum* conidia suspension (*F. graminearum* isolate IFA66) was prepared as described in Schweiger et al. (2016). Heads were inoculated individually when they were flowering by spraying three milliliters of a *F. graminearum* inoculum (spore concentration of 5 × 10^4^ ml^−1^ and 1 ml Tween 20 per liter) using a hand-held sprayer. Inoculated heads were covered with translucent polyethylene bags for 30 h assuring high humidity for optimal fungal development. AR was evaluated on five basal florets per inoculated head five days after inoculation (dai). A floret was scored as retained when at least one anther remained inside the floret or was trapped between lemma and palea. Number of infection sites per inoculated head was assessed at 8, 12 and 16 days after inoculation. Infections sites are defined as clearly separated starting points of Fusarium infection within a head. Number of diseased heads and number of symptomatic spikelets per spike were assessed at 8, 12, 16, 20 and 24 dai. The total number of spikelets of inoculated heads was counted. The percentage of symptomatic spikelets over the total number of inoculated spikelets per scoring date was calculated and used to determine the area under the disease progress curve (AUDPC) as described by Buerstmayr et al. (2000). AR was expressed in percent of florets with at least one anther retained. Number of inoculated heads per pot ranged from 10 to 22, with an average of 14 inoculated heads per pot. Plant height per pot was measured in cm. Each pot was considered as an experimental unit. Scoring data were averaged per pot over the total number of inoculated heads.

#### Field experiments

All field experiments were conducted at IFA Tulln, Austria (16^o^04,16′E, 48^o^19,08′N, 177 m above sea level) with four replicates per genotype and year. Lines of the *Rht1* NIL groups RE-NIL1, CM, FRxRE and MI along with their recurrent parents were tested in years 2019, 2020 and 2021 and RE and CM QTL-by-Rht1 NILs were tested in 2020 and 2021. Within each replication, NILs with the same near-isogenic background (including recurrent parents) were organized into semi-dwarfing and tall subgroups that were planted next to each other. NILs within tall and dwarf subgroups were fully randomized. This field design aimed to minimize potential height effects of neighboring plots. Plots consisted of double rows of 1 m length with 17-cm spacing. Sowing time was early spring in all years. Artificial spray inoculation started when the first plot reached mid-anthesis and was repeated every other day until 2 days after the last plot reached mid-anthesis. About 100 ml m^−2^ of freshly diluted macroconidia suspension (spore concentration of 2.5 × 10^4^ ml^−1^) of the *F. culmorum* single-spore isolate ‘Fc91015’, prepared as described by Buerstmayr et al. (2000), was sprayed onto the heads using a battery-driven backpack sprayer. Inoculations were carried out in the late afternoons from 4:00 to 6:00 p.m. An automated mist-irrigation system triggered by leaf wetness measurements was used to ensure high humidity during the first 20 h after each inoculation. FHB incidence scoring was assessed individually per plot 21 days after mid-flowering as the percentage of heads showing ≥ 1 symptomatic spikelets out of 40 and 50 randomly chosen heads in 2019 and 2021, respectively. FHB severity was visually estimated as the percentage of infected spikelets within each plot at 10, 14, 18, 22 and 26 days after anthesis and used for calculating the AUDPC as described by Buerstmayr et al. (2000). The percentage of retained anthers was assessed five days after anthesis on 30 florets by inspecting three basal florets of ten randomly chosen heads. Plant height (cm) was measured in all plots of all years.

#### Phenotypic data analysis

Statistical analysis was performed in R version 4.0.3 (R Core Team 2020) and was done for each NIL group and for field and greenhouse experiments separately. Variance components were determined by the restricted maximum likelihood (REML) method, assuming a general linear mixed model using the R package lme4 (Bates et al. 2015):

For *Rht1*-NIL groups, RE-NIL1, CM, FRxRE and MI:

$$Y_{{{\text{ikl}}}} = \mu + t_{i} + e_{k} + te_{ik} + b_{l} \left( {e_{k} } \right) + \varepsilon_{{{\text{ikl}}}} .$$

where *μ* denotes the overall mean, *t*_*i*_ the effect of *Rht1* haplotype *i*, *e*_*k*_ the effect of the experiment *k*, *te*_ik_ the interaction between *Rht1* haplotype *i* and experiment *k*, *b*_*l*_(*e*_*k*_) the effect of the block *l* nested in the experiment *k*, and *ε* the residual effect.

For RE and CM QTL-by-Rht1 groups:

$$Y_{{{\text{ijkl}}}} = \mu + t_{i} + q_{j} + tq_{{{\text{ij}}}} + e_{k} + r_{l} \left( {e_{k} } \right) + te_{{{\text{ik}}}} + qe_{{{\text{ik}}}} + tqe_{{{\text{ijk}}}} + \varepsilon_{{{\text{ijkl}}}}$$

where *μ* denotes the overall mean, *t*_*i*_ the effect of *Rht1* haplotype *i*, *q*_*j*_ the effect of *QTL* haplotype *j*, *tq*_ij_* the* interaction between *Rht1* haplotype *i* and QTL haplotype *j*, *e*_*k*_ the effect of the experiment *k*, *te*_ik_ the interaction between *Rht1* haplotype *i* and experiment *k*, *tqe*_ijk_ the interaction between *Rht1* haplotype *i, QTL* haplotype *j* and experiment *k*, *r*_*l*_(*e*_*k*_) the effect of the replication *l* nested in the experiment *k*, and *ε*_ijkl_ the residual effect. *Rht1* and *QTL* haplotypes and QTL-by-Rht1 interaction were considered as fixed effects, all other factors were considered as random effects.

Contrasts between haplotypic groups were assessed with the glht function (method: Tukey, *p* adjustment: Bonferroni) of the multcomp package (Hothorn et al. 2008).

## Results

### Effects of *Rht1* haplotypes on plant height, anther retention and FHB resistance in the *Rht1*-NIL groups RE-NIL1, CM, FRxRE and MI

*Rht1* haplotype had a strong and significant effect on plant height in all *Rht1-*NIL groups in greenhouse and field experiments (Table S1). The *Rht1* haplotype effect on AR was significant in greenhouse experiments for groups RE-NIL1, CM and FRxRE and in fields experiments for RE-NIL1, CM and MI and marginally significant in the green house for MI (*p* = 0.052) and in field tests for FRxRE (*p* = 0.086). The impact of *Rht1* haplotype on FHB incidence and AUDPC was significant in all NIL groups under field conditions. *Rht1* haplotypes had a significant effect in greenhouse experiments on AUDPC and on the number of infection sites in group RE-NIL1. *Rht1* significantly influenced the number of infection sites in group FRxRE and had a weak impact in group MI (*p* = 0.062) under greenhouse conditions.

### Effects of FHB resistance QTL *Fhb1* and *Qfhs.ifa-5A* in combination with the *Rht1* genes on plant height, anther retention and FHB resistance

Analysis of variance revealed a significant effect of the *Rht1* haplotype on plant height, AR%, FHB incidence and AUDPC in group RE and on plant height and AUDPC in group CM. The status of the FHB resistance alleles significantly affected AR%, FHB incidence and AUDPC in group RE, and AR% and AUDPC in group CM. Significant QTL-by-Rht1 interaction was revealed for plant height in group CM and for FHB incidence and AR% in group RE and CM (Table S2).

### Comparing the effect of the *Rht1* haplotype on plant height, AR and FHB resistance traits

#### Plant height

**Fig. 1:**
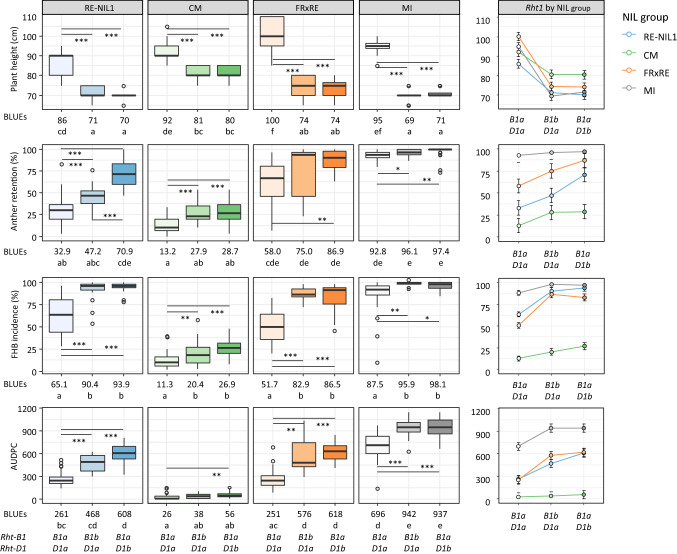
Boxplots of near isogenic lines (NILs) grouped by *Rht1* allele status for the recurrent NIL groups RE-NIL1, CM, FRxRE and MI for plant height, anther retention, FHB incidence, and area under the disease progress curve (AUDPC). Medians are indicated by solid bold lines and outliers by open circles. Comparisons are based on best linear unbiased estimators (BLUEs) across field trials. Bonferroni correction was used to adjust for multiple pairwise contrasts. Different letters below BLUEs indicate significant differences among NIL groups (*p* < 0.05), and bars between boxes indicate significant differences among *Rht1* haplotypes within NIL groups. Signif. codes: ***p < 0.001; **0.001 < *p* < 0.01; *0.01 < *p* < 0.05. Interaction plots depict BLUEs and standard errors of *Rht1* haplotypes by NIL group interaction

**Fig. 2:**
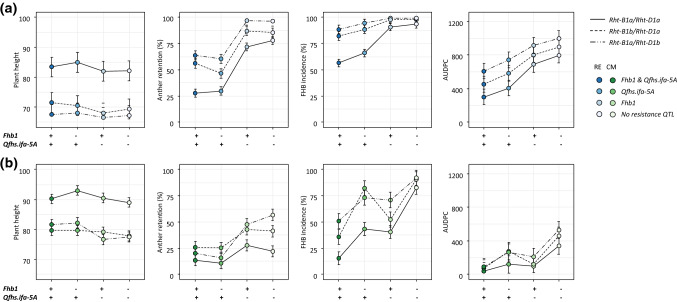
Interaction plots depict best linear unbiased estimators (BLUEs) and standard errors for plant height, anther retention, FHB incidence and area under the disease progress curve (AUDPC) for combinations of *Rht1* haplotypes and FHB resistance QTL within **a** RE and **b** CM QTL-by-Rht1 NIL groups

Wild-type lines (*B1aD1a*) of group FRxRE had the tallest plant phenotype (≥ 100 cm) and were significantly taller than wild-type lines of groups RE-NIL1 (90 cm) and MI (88 cm) and of groups RE-NIL1 (86 cm) and CM (92 cm) in greenhouse and field experiments, respectively (Figs. 1, S1; Tables 3, S3). Plant height reduction of introgressed dwarfing alleles was significant in all four recurrent NIL groups and was highest in FRxRE NILs, followed by MI, and RE-NIL1, and was lowest in CM (Figs. 1, S1; Table 3). The *Rht1* genes similarly effected plant height among the RE and CM QTL-by-Rht1 groups. Wild-type *Rht1* haplotypes of all FHB resistance QTL combinations were consistently taller than their corresponding *Rht-B1b* or *Rht*-*D1b* NILs (Figs. 2, S2, S3; Table 4). *Rht-B1b* and *Rht-D1b* dwarfing alleles led to similar decreases in height within NIL groups.

**Table 3:** Best linear unbiased estimators (BLUEs) across greenhouse experiments of NILs grouped by NIL group and *Rht1* haplotype combination for plant height (PH (cm)), anther retention (AR (%)), number of infection sites per head (№ IS) and area under the disease progress curve (AUDPC)

NIL group	*Rht1* haplotype	PH (cm)^a^		AR (%)^a^		№ IS^a^		AUDPC^a^	
		BLUEs		BLUEs		BLUEs		BLUEs	
RE-NIL1	*B1aD1a*	90	e	30.7	a	0.7	bc	642	ab
RE-NIL1	*B1bD1a*	67	ab	62.7	cd	1.0	cd	1356	b
RE-NIL1	*B1aD1b*	65	ab	77.4	de	1.2	d	1610	b
CM	*B1aD1a*	97	f	36.6	ab	0.1	a	30	a
CM	*B1bD1a*	80	d	56.8	bd	0.2	a	38	a
CM	*B1aD1b*	77	cd	55.9	bd	0.1	a	37	a
FRxRE	*B1aD1a*	103	f	54.4	bc	0.2	a	325	a
FRxRE	*B1bD1a*	69	ac	80.2	de	0.4	ab	905	ab
FRxRE	*B1aD1b*	71	bc	87.8	e	0.4	ab	726	ab
MI	*B1aD1a*	88	e	87.6	e	1.0	d	754	ab
MI	*B1bD1a*	62	a	91.7	e	1.2	d	1024	ab
MI	*B1aD1b*	62	a	97.6	e	1.2	d	1008	ab

^a^BLUEs of groups with different letters are significantly different (Bonferroni *p*-value adjustment, *p* < 0.05)

**Table 4:** Best linear unbiased estimators (BLUEs) across experiments of RE and CM QTL-by-Rht1 NILs grouped by *Rht1* haplotype and FHB resistance QTL combination

Trait	*Rht1* haplotype	FHB resistance QTL		NIL group RE		NIL group CM	
				BLUEs^a^		BLUEs^a^	
Plant height	*B1aD1a*	*Fhb1*	*Qfhs.ifa-5A*	83.4	b	90.2	b
	*B1aD1a*	*–*	*Qfhs.ifa-5A*	85.0	b	93.0	b
	*B1aD1a*	*Fhb1*	–	82.0	b	90.5	b
	*B1aD1a*	–	–	82.2	b	89.0	b
	*B1bD1a*	*Fhb1*	*Qfhs.ifa-5A*	71.6	a	79.7	a
	*B1bD1a*	–	*Qfhs.ifa-5A*	70.6	a	79.7	a
	*B1bD1a*	*Fhb1*	–	68.1	a	79.1	a
	*B1bD1a*	–	–	69.4	a	77.8	a
	*B1aD1b*	*Fhb1*	*Qfhs.ifa-5A*	67.5	a	81.6	a
	*B1aD1b*	–	*Qfhs.ifa-5A*	68.1	a	82.0	a
	*B1aD1b*	*Fhb1*	–	66.6	a	76.6	a
	*B1aD1b*	–	–	67.2	a	77.5	a
Anther retention	*B1aD1a*	*Fhb1*	*Qfhs.ifa-5A*	27.8	a	13.4	a
	*B1aD1a*	–	*Qfhs.ifa-5A*	29.8	a	10.7	a
	*B1aD1a*	*Fhb1*	–	71.9	cde	27.7	abc
	*B1aD1a*	–	–	78.0	def	22.0	ab
	*B1bD1a*	*Fhb1*	*Qfhs.ifa-5A*	56.2	bc	26.0	abc
	*B1bD1a*	–	*Qfhs.ifa-5A*	46.5	ab	25.2	abc
	*B1bD1a*	*Fhb1*	–	87.1	eg	43.0	bd
	*B1bD1a*	–	–	85.3	eg	41.3	bd
	*B1aD1b*	*Fhb1*	*Qfhs.ifa-5A*	63.6	bcd	20.0	ab
	*B1aD1b*	–	*Qfhs.ifa-5A*	60.3	bcd	15.6	a
	*B1aD1b*	*Fhb1*	–	96.9	g	47.7	cd
	*B1aD1b*	–	–	96.3	fg	56.7	d
FHB incidence	*B1aD1a*	*Fhb1*	*Qfhs.ifa-5A*	56.5	a	15.4	a
	*B1aD1a*	–	*Qfhs.ifa-5A*	65.9	a	43.3	b
	*B1aD1a*	*Fhb1*	–	90.8	bc	40.6	b
	*B1aD1a*	–	–	93.4	bc	82.5	d
	*B1bD1a*	*Fhb1*	*Qfhs.ifa-5A*	82.3	b	35.8	ab
	*B1bD1a*	–	*Qfhs.ifa-5A*	88.5	bc	82.3	d
	*B1bD1a*	*Fhb1*	–	98.0	c	52.3	bc
	*B1bD1a*	–	–	97.8	c	90.5	d
	*B1aD1b*	*Fhb1*	*Qfhs.ifa-5A*	88.5	bc	51.0	bc
	*B1aD1b*	–	*Qfhs.ifa-5A*	94.3	bc	73.3	cd
	*B1aD1b*	*Fhb1*	–	99.0	c	71.0	cd
	*B1aD1b*	–	–	99.0	c	92.0	d
AUDPC	*B1aD1a*	*Fhb1*	*Qfhs.ifa-5A*	300.7	a	40.6	a
	*B1aD1a*	–	*Qfhs.ifa-5A*	406.6	ab	119.2	ac
	*B1aD1a*	*Fhb1*	–	687.9	cd	95.5	ab
	*B1aD1a*	–	–	796.8	cf	339.6	ac
	*B1bD1a*	*Fhb1*	*Qfhs.ifa-5A*	454.1	ab	70.9	ab
	*B1bD1a*	–	*Qfhs.ifa-5A*	584.3	bc	276.8	ac
	*B1bD1a*	*Fhb1*	–	804.4	cf	123.7	ac
	*B1bD1a*	–	–	896.1	df	459.6	bc
	*B1aD1b*	*Fhb1*	*Qfhs.ifa-5A*	609.0	bc	91.6	ab
	*B1aD1b*	–	*Qfhs.ifa-5A*	741.4	cde	260.3	ac
	*B1aD1b*	*Fhb1*	–	915.9	ef	206.8	ac
	*B1aD1b*	–	–	997.3	f	528.9	c

^a^BLUEs of groups with different letters are significantly different (Bonferroni *p*-value adjustment, *p* < 0.05)

#### Anther retention

Average AR% of wild-type haplotypes in *Rht1-*NIL groups RE-NIL1 (31% or 33%) and CM (37% or 13%) was markedly lower than in FRxRE (54% or 58%) and was highest in MI (88% or 93%) in respective greenhouse or field experiments. There were more anthers retained in dwarfing NILs in field and greenhouse experiments in all groups, but the difference between tall and semi-dwarfing *B1b* NILs was below significance in field experiments of NIL group FRxRE and in greenhouse experiments of NIL group MI (Figs. 1, S1). The *Rht1-D1b* allele had a significantly stronger effect compared to the *Rht-B1b* allele in group RE-NIL1, while in the groups CM, FRxRE and MI no differences were observed between *B1b* and *D1b* isolines. The increase in AR in the dwarfed versus tall lines was highest in group RE-NIL1, followed by FRxRE and CM and was smallest in group MI. However, since the tall MI haplotypes had already nearly complete anther retention, there was little room for further increase. The *Rht1* haplotype in RE and CM QTL-by-Rht1 group similarly affected AR%, whereby higher proportions of anthers were retained in semi-dwarfing NILs compared to their tall variants and level of AR% were much higher in RE compared to CM background (Figs. 2, S2, Table 4).

#### FHB resistance traits

Generally, within QTL-by-Rht1 and *Rht1*-NIL groups in most cases no significant differences in FHB susceptibility were found between the semi-dwarfing *Rht-B1b* and *Rht-D1b* haplotypes, although there was a trend toward higher susceptibility for *D1b* than for *B1b* carriers (Figs. 1, 2, S3). Differences between tall and dwarf lines were more pronounced under field conditions and were particularly high among NILs of groups RE-NIL1 and FRxRE (Fig. 1). *Rht1* haplotype did not affect FHB resistance level of CM and MI NILs in the greenhouse and had a relatively small impact under field conditions (Figs. 1, S1; Table 3). The effect of the dwarfing alleles was low in the highly FHB-resistant CM background, and the dwarfed NILs remained highly resistant in greenhouse and field experiments. FHB severity in all MI NILs was high, regardless of height. The FHB severity levels in MI NILs were particularly high under field conditions. Infection of FRxRE NILs was relatively low in the greenhouse but high in the field. Tall variants of RE and CM QTL-by-Rht1 groups were more resistant than their corresponding dwarf genotypes among subgroups of all FHB resistance QTL combinations, and these differences were in most cases significant (Fig S3, Table 4).

### Comparing the effect of the FHB resistance QTL and *Rht1* combination on plant height, AR and FHB resistance traits

#### Plant height

Only minor height differences were observed between NILs with different QTL combinations.

#### Anther retention

AR was strongly influenced by the allele status at the *Qfhs.ifa-5A* resistance QTL, while *Fhb1* had no effect on AR (Figs. 2, S2, S3; Table 4). The resistance alleles at *Qfhs.ifa-5A* clearly reduced AR in tall as well as semi-dwarf haplotypes. Differences were particularly high and always significant between *Qfhs.ifa-5A* carriers and non-carriers within *Rht1* subgroups of RE background, while the differences were smaller in CM NILs and only significant within the tall and the semi-dwarfing *Rht-D1b* haplotypes (Fig S2). Tall NILs without the *Qfhs.ifa-5A* resistance allele had 10–30% more anthers retained than semi-dwarfing NILs with the *Qfhs.ifa-5A* resistance allele.

#### *FHB resistance *traits

The introgression of *Qfhs.ifa-5A* alone or in combination with the *Fhb1* resistance allele into the RE background decreased FHB incidence and AUDPC in tall and dwarf haplotypes (Figs. 2, S2). The ranking of FHB resistance QTL combinations within *Rht1* subgroups was constant whereby NILs with *Qfhs.ifa-5A* and *Fhb1* were most resistant, followed by NILs containing *Qfhs.ifa-5A* only, and NILs carrying *Fhb1* alone were almost as susceptible as NILs without any resistance QTL. Differences between QTL subgroups were larger within the tall *Rht1* lines (Figs. 2, S2; Table 4). QTL subgroups that had both QTL introgressed were usually significantly more resistant than lines containing solely *Fhb1* or no resistance QTL but were in most cases not different from NILs which contained only the *Qfhs.ifa-5A* resistance QTL (Fig S2; Table 4). Semi-dwarfing NILs containing the *Qfhs.ifa-5A* QTL alone or in combination with *Fhb1* were consistently slightly more resistant than tall variants without the *Qfhs.ifa-5A* QTL. Rank order of QTL combination within *Rht1* subgroups of CM NILs was consistent but differed slightly from rank order of RE NILs. CM NILs having both QTL combined performed best, second were *Fhb1*, closely followed by *Qfhs.ifa-5A* NILs, and lines without a resistance QTL were most susceptible (Figs. 2, S2; Table 4). Resistance to FHB incidence was significantly better in lines with both QTL combined compared to NILs without a resistance QTL, and NILs containing solely *Fhb1* performed equally or better than NILs with *Qfhs.ifa-5A* only or no QTL (Fig S2). AUDPC remained generally low and was only significantly different in *Rht-D1b* subgroups between NILs with both resistance QTL and NILs with no resistance QTL.

## Discussion

### Effects of *Rht1* haplotypes on plant height, anther retention and FHB resistance

The association between increased FHB susceptibility and shorter plant height has been observed many times, leaving little doubt about the causal connection between plant height and FHB resistance. However, plant height ‘per se’ may not be the only driving factor, since for many plant height QTL no effect on FHB resistance has been reported (Buerstmayr et al. 2020). FHB is a floral disease and flowering-related traits such as the amount of retained or extruded anthers (Buerstmayr et al. 2020; Kubo et al. 2013; Steiner et al. 2019), and the duration and the width of the opening of the florets during anther extrusion have been associated with FHB resistance (Gilsinger et al. 2005). Several independent studies identified an overlap of the *Rht1* loci with QTL for anther extrusion and for FHB resistance (Buerstmayr and Buerstmayr 2016; He et al. 2016; Lu et al. 2013; Xu et al. 2020; Zhu et al. 2021). Reduced plant height in combination with increased AR is thus particularly disadvantageous for FHB resistance and may explain why the *Rht1* loci regularly coincided with QTL for FHB resistance, where the *Rht1* loci commonly showed the strongest effect among the identified FHB QTL (Buerstmayr et al. 2020). Our analysis revealed an increase in AR in semi-dwarfing lines of all NIL groups (Fig. 1, S1; Table 3). Effect size of the semi-dwarfing alleles on AR, however, depended on the genetic background and was highest in RE-NIL1, followed by FRxRE and CM, and was lowest in MI. We also observed a stronger increase in AR% in *Rht-D1b* compared to *Rht-B1b* lines within group RE-NIL1 (Figs. 1, S1; Table 3). A genotype-dependent effect of *Rht-B1* alleles on anther extrusion was also reported by Okada et al. (2019), but unlike our results, they reported that the *Rht-D1b* allele did not change anther extrusion.

The strongest increase in susceptibility was observed in the semi-dwarfing NILs of group RE-NIL1 and FRxRE, followed by MI. This rank order largely corresponded with the relative increase in AR%, which was also highest in RE-NIL1, followed by FRxRE and MI. FHB susceptibility among CM NILs was largely unaffected by the introgression of dwarfing alleles despite significantly more retained anthers in the shorter NILs. The higher proportion of retained anthers in *Rht-D1b* compared to the *Rht-B1b* lines in group RE-NIL1 is also reflected in a higher susceptibility of the *Rht-D1b* lines. Since RE-NIL1 *Rht-D1b* and *Rht-B1b* NILs have approximately equal plant height, we assume that the difference in FHB susceptibility is directly related to the differences in AR%. This supports our earlier findings in a double haploid population, where the *Rht-D1b* allele had a significantly greater impact on AR and FHB severity than the *Rht-B1b* allele (Buerstmayr and Buerstmayr 2016).

We expect only a small (if any) effect of plant height on FHB resistance in our greenhouse experiments*.* Most external factors that may play a role in field tests and passively influence FHB resistance in a plant-height-dependent manner, for instance wind, morning or evening dew, or rain-splashed spores, can be excluded under controlled greenhouse conditions. The influence of soil humidity derived from greenhouse pots is limited compared to conditions in field tests. The observed changes in resistance through introgression of semi-dwarfing *Rht-B1b* or *Rht-D1b* alleles may thus, at least under greenhouse conditions, primarily reflect the changes in AR%. However, we cannot rule out additional pleiotropic effects. Saville et al. (2012) proposed that DELLA proteins have a pleiotropic effect on disease resistance by altering stress response and controlled cell death, with DELLA accumulating semi-dwarfing *Rht1* lines being more susceptible to initial infection (type 1 resistance) but more resistant to the later colonization phase (type 2 resistance).

### Effects of FHB resistance QTL-by-Rht1 haplotype combinations on plant height, anther retention and FHB resistance within the highly susceptible RE and the highly resistant CM background

The allele status at the *Rht1* genes and at the *Qfhs.ifa-5A* QTL affected AR, whereby the *Qfhs.ifa-5A* resistance allele reduced AR to at least the same level as the semi-dwarfing alleles increased it (Figs. 2, S2, Table 4). The proportion of retained anthers had a large impact on FHB resistance, accordingly absence of the *Qfhs.ifa-5A* resistance as well as the presence of dwarfing alleles increased FHB susceptibility. Retained anthers as potential susceptibility factors were particularly relevant in RE lines where AR% was generally higher than in CM lines, changes in FHB resistance due to changes in allele status at the *Rht1* or *Qfhs.ifa-5A* genes were higher in the RE than in the CM NILs.

There is clear evidence of a negative effect of retained anthers on FHB resistance, whereby the absence of anthers enhances resistance to initial infection but does not protect plants from fungal spreading within spikes (Steiner et al. 2019). FHB infection starts inside the floral cavities, and anthers partially trapped between the palea and lemma are important entry points for the fungus into the florets (Kang and Buchenauer 2000). Once the spores have successfully entered the florets, the floral cavities provide conditions that are highly conducive for pathogen development. Spore germination and hyphal colonization were much more enhanced on anthers, pollen and stigma compared to the surrounding tissues of the lemma and palea and were the preferred tissue at the onset of FHB infection (Kang and Buchenauer 2000; Miller et al. 2004; Pugh 1933). Successful anther extrusion depends on a multitude of genes that may regulate different aspects of the flowering process; for instance, lodicule swelling, anther dehiscence, filament elongation, rigidity of the filaments, flower opening angle and flower opening duration, but it may also be influenced by spike architecture or the shape of the glumes. The complex nature of anther extrusion likely explains the observed background dependence of the *Rht1* genes and the *Qfhs.ifa-5A* genes on modulating AR.

Gene expression analysis revealed a stress response NAC SECONDARY WALL THICKENING PROMOTING FACTOR1 (*NST1*)-like protein as a potential candidate gene for *Qfhs.ifa-5AS* (Buerstmayr et al. 2021). Genetic experiments in the model plant *Arabidopsis thaliana* showed that *NST1* is key regulator of secondary cell wall biosynthesis in anther endothecium cells (Mitsuda et al. 2005, 2007). *NST1* also induced ectopic secondary wall thickening in various tissues, including filaments of stamens (Mitsuda et al. 2005); however, it is unknown if this has an influence on anther extrusion.

*Rht1* genes control cell elongation; one may thus expect shorter stamen filaments as demonstrated in Arabidopsis (Cheng et al. 2004) or changes in tissue structures due to more compact cell types. However, in a detailed search for optimal pollinator traits no association between *Rht-D1* status and anther filament length was observed, but anther length was significantly affected (Okada et al. 2021).

*Fhb1* had no influence on AR and had only a very small impact on FHB resistance in the susceptible RE background, whereas *Fhb1* was at least as good as *Qfhs.ifa-5A* improving FHB incidence in the highly resistant CM background. CM NILs without any resistance QTL reached FHB incidence levels that were almost as high as those in the RE NILs; however, the respective AUDPC scores did not raise to the same level. This suggests that the genetic background of CM confers, in addition to *Fhb1*, excellent type 2 resistance that is able to block the fungus from spreading within the spike even under high disease pressure.

## Conclusion

The current study confirms earlier published results that semi-dwarfing alleles have a negative impact on FHB resistance. The data shed new light on the dependence of this association on the genetic background. Studying NILs with greatly different background resistance illustrates that a highly resistant line, such as CM-82036, retains a high resistance level even if semi-dwarf *Rht1* alleles are introduced. A highly susceptible line, such as Michael, is susceptible almost regardless of its *Rht1* status. Introducing semi-dwarfing alleles into a moderately resistant line, such as RE-NIL1, simultaneously leads to a significant increase in anther retention and FHB susceptibility. The well-known QTL *Qfhs.ifa-5A* is capable of increasing FHB resistance and anther extrusion as efficiently as semi-dwarfing alleles (*Rht-D1b* or *Rht-B1b*) decrease these traits. Thus introducing *Qfhs.ifa-5A* into semi-dwarf breeding populations is highly recommended in order to lay a baseline for safeguarding FHB resistance in wheat. Adding *Fhb1* on top will frequently lead to an even better level of FHB resistance and is therefore recommended for wheat breeding where FHB resistance is considered a key trait.

## Supplementary Information

Below is the link to the electronic supplementary material.

Supplementary file1 (PDF 270 KB)

Supplementary file2 (PDF 463 KB)

## Data Availability

The plant material and datasets employed in this study are available from the corresponding author on reasonable request.
