# Supplementary material for: The effect of the Rht1 haplotype on Fusarium head blight resistance in relation to type and level of background resistance and in combination with Fhb1 and Qfhs.ifa-5A
Source: Theor Appl Genet. 2022 Apr 9;135(6):1985–96. doi: 10.1007/s00122-022-04088-x (PMC9205817; doi:10.1007/s00122-022-04088-x)
Supplement: Supplementary file 1 — Supplementary file1 (PDF 270 KB) [file 122_2022_4088_MOESM1_ESM.pdf]

Online Resource 1

Article title: The effect of the *Rht1* haplotype on Fusarium head blight resistance in relation to type and level of background resistance and in combination with *Fhb1* and *Qfhs.ifa-5A*

Journal: Theoretical and applied genetics

Authors: Maria Buerstmayr, Hermann Buerstmayr

Name, affiliation, and email of corresponding author:

Maria Buerstmayr, Department for Agrobiotechnology Tulln, BOKU-University of Natural Resources and Life Sciences-Vienna, Konrad Lorenz Str. 20, 3430 Tulln, Austria
e-mail: maria.buerstmayr@boku.ac.at

Content: Table S1 – Table S3

Table S1 Analysis of variance table for *Rht1*-NIL groups RE-NIL1, CM, FRxRE and MI across greenhouse and across field experiments

Table S2 Analysis of variance table of RE and CM QTL-by-Rht1 groups across field experiments

Table S3 Best linear unbiased estimators (BLUEs) across field experiments of NILs grouped by NIL group and *Rht1* haplotype for plant height (PH (cm)), anther retention (AR(%)), Fusarium head blight (FHB) incidence and area under the disease progress curve (AUDPC)

Table S1 Analysis of variance table for *Rht1*-NIL groups RE-NIL1, CM, FRxRE and MI across greenhouse and across field experiments

		Green house experiments			Field experiments		
Trait	NIL group	Mean square[a]	*F* value	*p* value	Mean square[a]	*F* value	*p* value
Plant height (cm)							
	RE-NIL1	2201.4	298.6	0.001	226.3	57.5	0.001
	CM	1930.7	116.3	< 2.2e-16	275.0	21.9	0.002
	FRxRE	6412.7	212.8	< 2.2e-16	1022.6	65.3	0.001
	MI	4187.7	497.2	< 2.2e-16	1107.1	186.5	0.000
Anther retention (%)							
	RE-NIL1	5065.6	43.1	0.005	9671.4	63.5	0.004
	CM	2298	17.1	0.000	988.2	16	0.011
	FRxRE	5010	20.2	0.003	536.1	5	0.086
	MI	422	3.2	0.052	213.6	7.6	0.025
Number of infection sites per head					FHB incidence (%)		
	RE-NIL1	1.38	17.2	0.000	7214.9	68.3	0.000
	CM	0.01	0.9	0.390	1660.1	17.8	0.000
	FRxRE	0.33	11.7	0.000	10717.0	93.4	< 2.2e-16
	MI	0.34	3.0	0.062	936.8	6.4	0.003
Area under the disease progress curve (AUDPC)							
	RE-NIL1	973509	7.8	0.035	249607	30.7	0.003
	CM	273	0.4	0.700	2644.6	6.4	0.045
	FRxRE	393173	3.6	0.110	72574.0	9.2	0.033
	MI	411761	2.9	0.260	270626	19.9	0.008

[a] Source of variance: *Rht1* haplotype

Table S2 Analysis of variance table of RE and CM *QTL*-by-*Rht1* groups across field experiments

	RE			CM		
Source	Mean square	*F* value	*p* value	Mean square	*F* value	*p* value
Plant height (cm)						
Rht1 haplotype	101.0	20.2	0.047	150.8	21.4	0.019
QTL haplotype	30.8	6.2	0.074	41.7	5.9	0.078
QTL-by-*Rht1* haplotype	9.9	2.0	0.070	35.3	5.0	0.000
Anther retention (%)						
Rht1 haplotype	17434.1	107.1	< 2.2e-16	1402.3	15.2	0.062
QTL haplotype	14946.4	91.8	0.001	3138.4	34.0	0.000
QTL-by-*Rht1* haplotype	532.6	3.3	0.004	371.3	4.0	0.034
FHB incidence (%)						
Rht1 haplotype	4607.3	55.0	< 2.2e-16	8861.2	38.6	0.000
QTL haplotype	3021.1	36.0	< 2.2e-16	14802.5	64.4	< 2.2e-16
QTL-by-*Rht1* haplotype	606.1	7.2	0.000	813.6	3.5	0.003
Area under the disease progress curve (AUDPC)						
Rht1 haplotype	435233	40.3	0.000	42493	8.4	0.107
QTL haplotype	387997	35.9	0.007	15418	3.0	0.192
QTL-by-*Rht1* haplotype	6273	0.6	0.738	8146	1.6	0.293

Table S3 Best linear unbiased estimators (BLUEs) across field experiments of NILs grouped by NIL group and *Rht1* haplotype for plant height (PH (cm)), anther retention (AR(%)), Fusarium head blight (FHB) incidence and area under the disease progress curve (AUDPC)

NIL group	*Rht1* haplotype	PH (cm)[a] BLUEs		AR (%)[a] BLUEs		FHB incidence[a] BLUEs		AUDPC[a] BLUEs	
RE-NIL1	*B1aD1a*	86	cd	33.1	ab	63.6	d	261	bc
RE-NIL1	*B1bD1a*	71	a	47.2	abc	90.4	ef	468	cd
RE-NIL1	*B1aD1b*	70	a	70.9	cde	93.9	ef	608	d
CM	*B1aD1a*	92	de	13.1	a	12.7	a	26	a
CM	*B1bD1a*	81	bc	27.9	ab	20.4	ab	38	ab
CM	*B1aD1b*	80	bc	28.7	ab	26.9	b	56	ab
FRxRE	*B1aD1a*	100	f	58.1	bd	50.7	c	251	ac
FRxRE	*B1bD1a*	74	ab	75.0	cde	86.5	ef	576	d
FRxRE	*B1aD1b*	74	ab	86.9	de	82.9	e	618	d
MI	*B1aD1a*	95	ef	92.9	de	88.2	ef	696	d
MI	*B1bD1a*	69	a	96.1	e	98.1	f	942	e
MI	*B1aD1b*	71	a	97.1	e	96.8	f	940	e

[a] BLUEs of groups with different letters are significantly different (Bonferroni *p*-value adjustment, $p < 0.05$)
