# Supplementary material for: The effect of the Rht1 haplotype on Fusarium head blight resistance in relation to type and level of background resistance and in combination with Fhb1 and Qfhs.ifa-5A
Source: Theor Appl Genet. 2022 Apr 9;135(6):1985–96. doi: 10.1007/s00122-022-04088-x (PMC9205817; doi:10.1007/s00122-022-04088-x)
Supplement: Supplementary file 2 — Supplementary file2 (PDF 463 KB) [file 122_2022_4088_MOESM2_ESM.pdf]

Online Resource 2

Article title: The effect of the *Rht1* haplotype on Fusarium head blight resistance in relation to type and level of background resistance and in combination with *Fhb1* and *Qfhs.ifa-5A*

Journal: Theoretical and applied genetics

Authors: Maria Buerstmayr, Hermann Buerstmayr

Name, affiliation, and email of corresponding author:

Maria Buerstmayr, Department for Agrobiotechnology Tulln, BOKU-University of Natural Resources and Life Sciences-Vienna, Konrad Lorenz Str. 20, 3430 Tulln, Austria
e-mail: maria.buerstmayr@boku.ac.at

Content: Fig. S1 – S3

Fig. S1 Boxplots of near isogenic lines (NILs) grouped by *Rht1* allele status for the recurrent *Rht1*-NIL groups RE-NIL1, CM, FRxRE and MI for plant height, anther retention, infection sites per head, and area under the disease progress curve (AUDPC) across greenhouse trials.

Fig. S2 Boxplots of NILs grouped by FHB QTL combination within *Rht1*-haplotype of **(A)** RE and **(B)** CM QTL-by-Rht1 NIL groups for plant height, anther retention, Fusarium head blight (FHB) incidence, FHB severity and area under the disease progress curve (AUDPC) .

Fig. S3 Boxplots of NILs grouped by *Rht1*-haplotype within FHB QTL combination of **(A)** RE and **(B)** CM QTL-by-Rht1 NIL groups for plant height, anther retention, Fusarium head blight (FHB) incidence, FHB severity and area under the disease progress curve (AUDPC) .

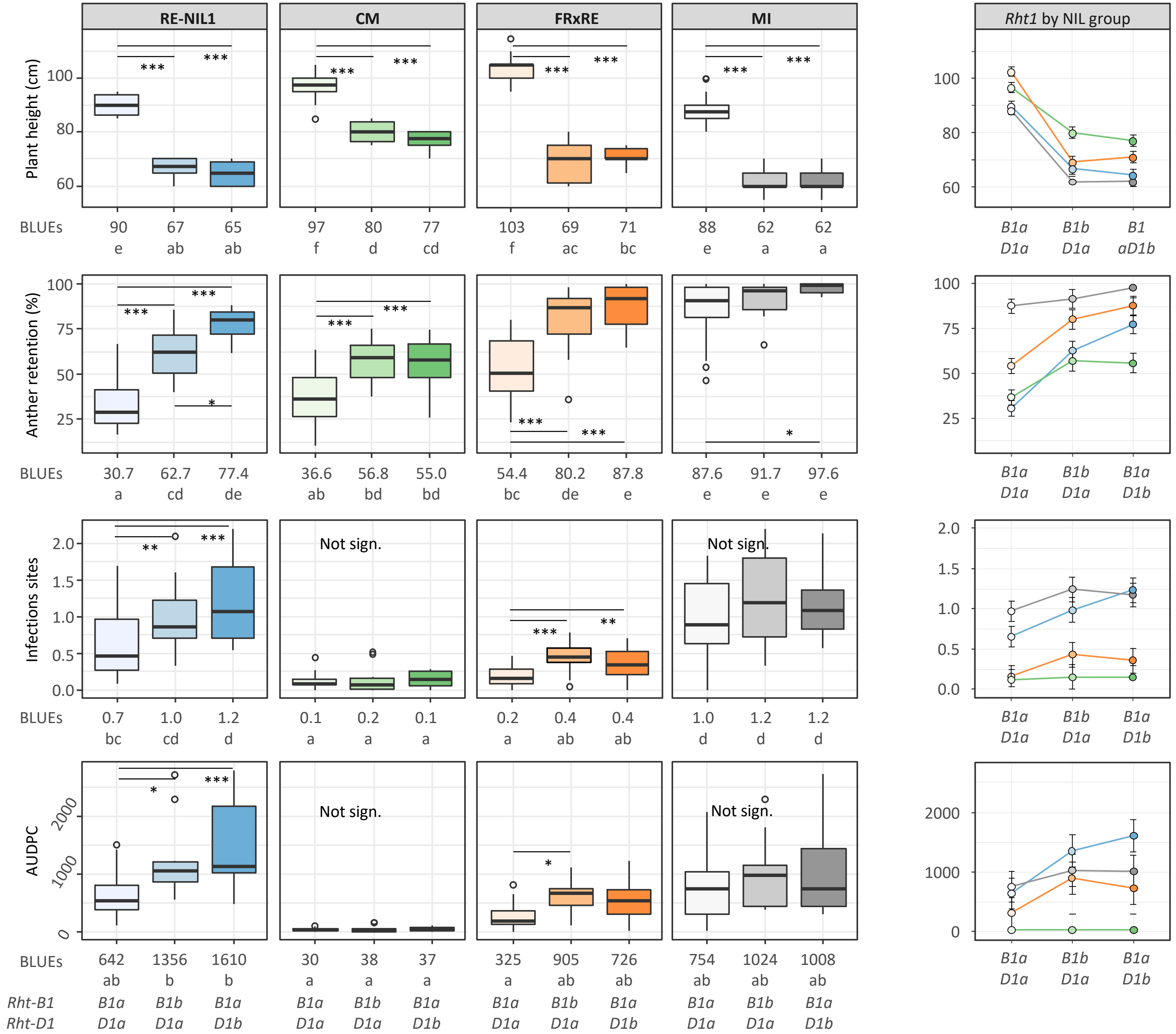

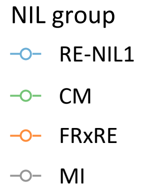

Fig. S1 Boxplots of near isogenic lines (NILs) grouped by *Rht1* allele status for the recurrent NIL groups RE-NIL1, CM, FRxRE and MI for plant height, anther retention, infection sites per head, and area under the disease progress curve (AUDPC). Medians are indicated by solid bold lines and outliers by open circles. Comparisons are based on best linear unbiased estimators (BLUEs) across greenhouse trials. Bonferroni correction was used to adjust for multiple pairwise contrasts. Different letters below boxes indicate significant differences among NIL groups ($p < 0.05$), and bars between boxes indicate significant differences among *Rht1* haplotypes within NIL groups. Signif. codes: '***' $p < 0.001$; '**' $0.001 < p < 0.01$; $0.01 < p <$ '*' < 0.05. Interaction plots depict BLUEs and standard errors of *Rht1* haplotypes by NIL group interaction

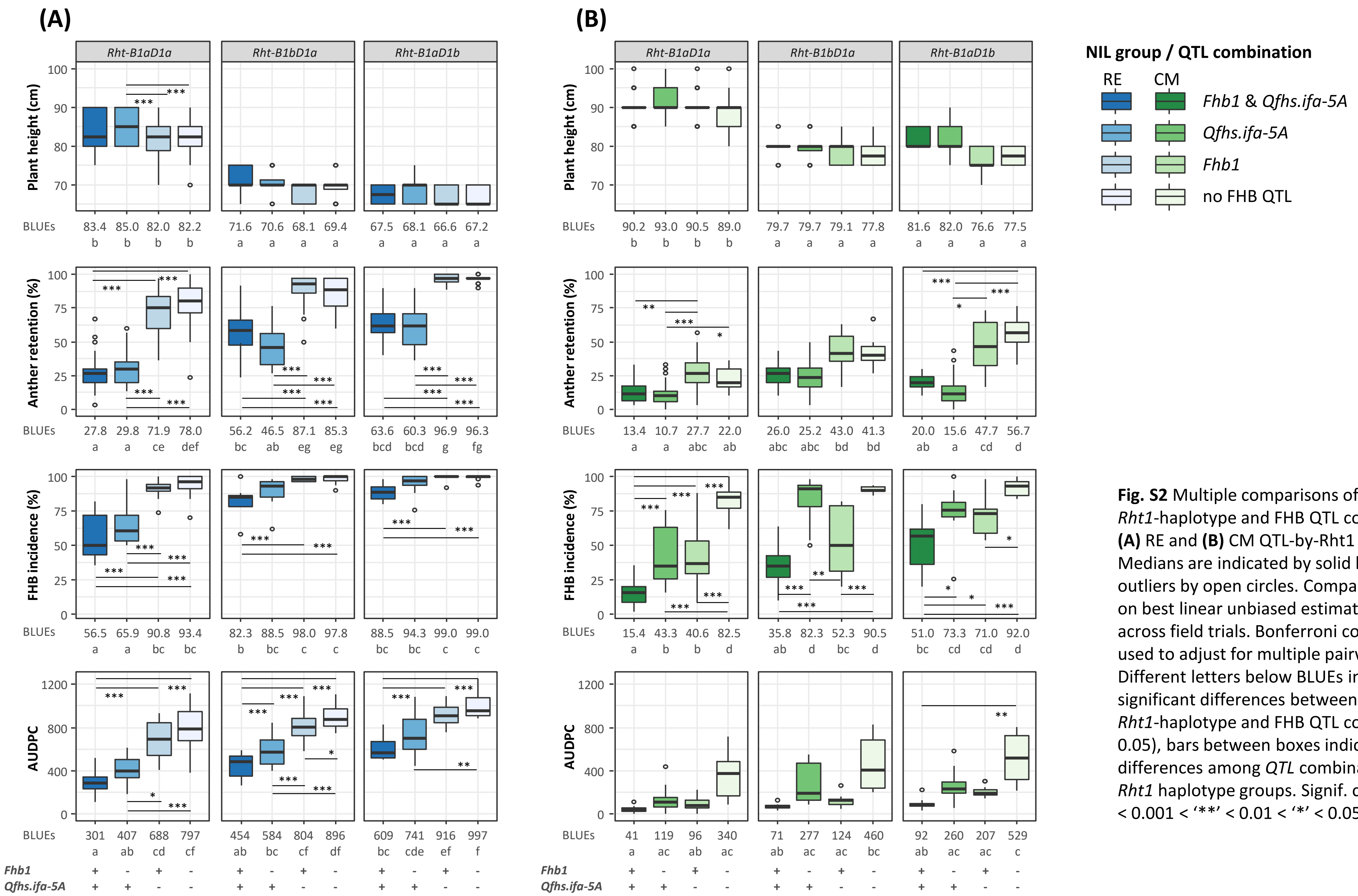

Fig. S2 Multiple comparisons of NILs grouped by *Rht1*-haplotype and FHB QTL combination of **(A)** RE and **(B)** CM QTL-by-Rht1 NIL groups. Medians are indicated by solid bold lines and outliers by open circles. Comparisons are based on best linear unbiased estimators (BLUEs) across field trials. Bonferroni correction was used to adjust for multiple pairwise contrasts. Different letters below BLUEs indicate significant differences between boxes among all *Rht1*-haplotype and FHB QTL combinations ($p < 0.05$), bars between boxes indicate significant differences among *QTL* combination within *Rht1* haplotype groups. Signif. codes: 0 < '***' < 0.001 < '**' < 0.01 < '*' < 0.05.

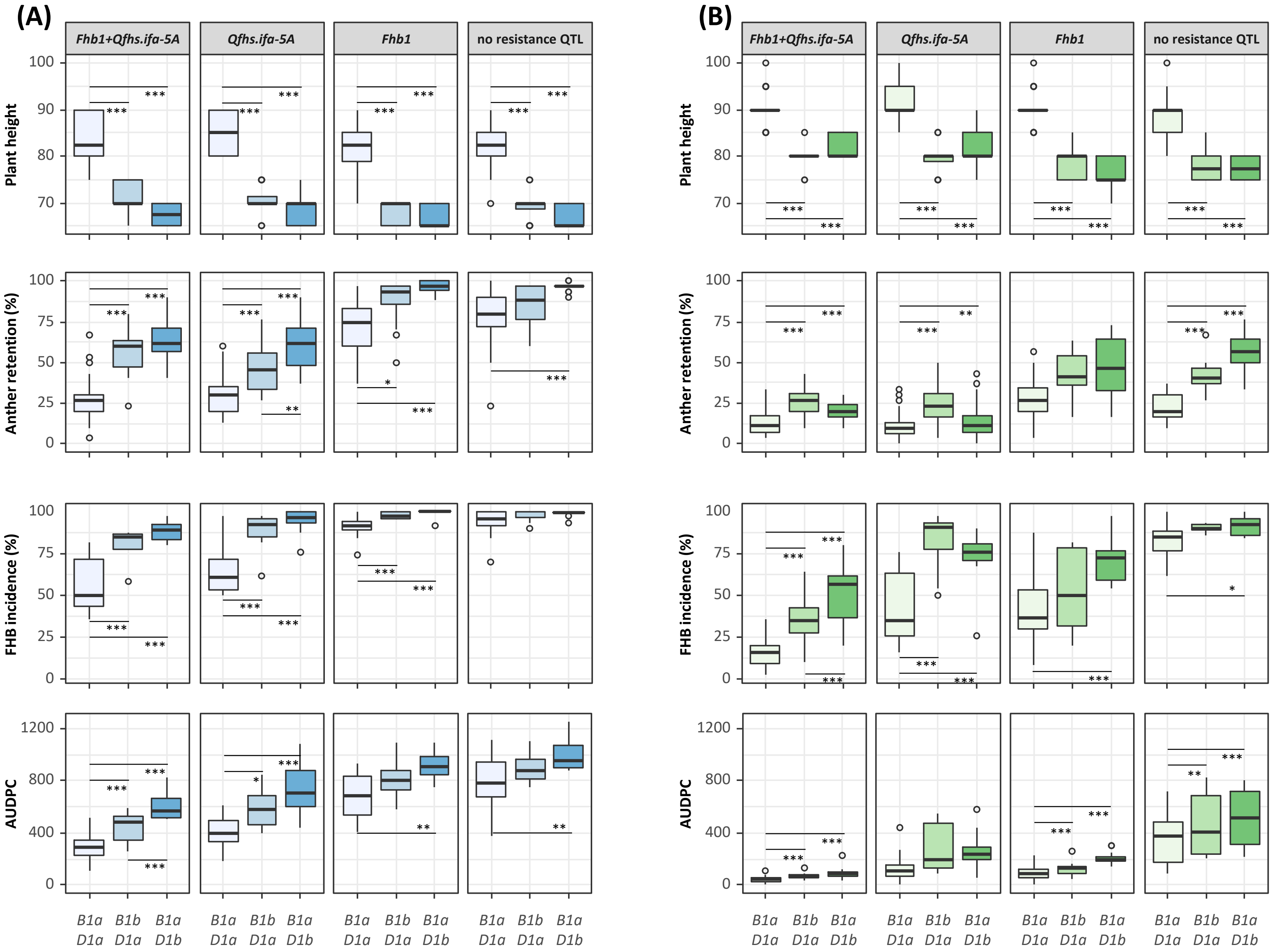

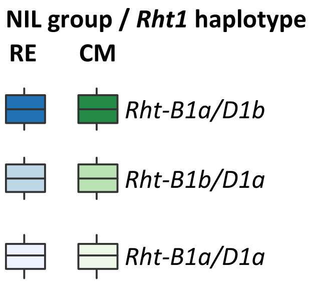

Fig. S3 Multiple comparisons of NILs grouped by *Qfhs.ifa-5A*, *Fhb1* and *Rht1* status of **(A)** RE and **(B)** CM QTL-by-Rht1 NIL group for plant height, anther retention, Fusarium head blight (FHB) incidence, FHB severity and area under the disease progress curve (AUDPC) illustrated by boxplots. Medians are indicated by solid bold lines and outliers by open circles. Comparisons are based on best linear unbiased estimators (BLUEs) across field trials. Bonferroni correction was used to adjust for multiple pairwise contrasts. Bars between boxes indicate significant differences among *Rht1* haplotypes within QTL subgroups. Signif. codes: 0 < '***' < 0.001 < '**' < 0.01 < '*' < 0.05.
